# A genetic approach for building different alphabets for peptide and protein classification

**DOI:** 10.1186/1471-2105-9-45

**Published:** 2008-01-24

**Authors:** Loris Nanni, Alessandra Lumini

**Affiliations:** 1 DEIS, Università di Bologna, Via Venezia 52, 47023 Cesena (FC), Italy

## Abstract

**Background:**

In this paper, it is proposed an optimization approach for producing reduced alphabets for peptide classification, using a Genetic Algorithm. The classification task is performed by a multi-classifier system where each classifier (Linear or Radial Basis function Support Vector Machines) is trained using features extracted by different reduced alphabets. Each alphabet is constructed by a Genetic Algorithm whose objective function is the maximization of the area under the ROC-curve obtained in several classification problems.

**Results:**

The new approach has been tested in three peptide classification problems: HIV-protease, recognition of T-cell epitopes and prediction of peptides that bind human leukocyte antigens. The tests demonstrate that the idea of training a pool classifiers by reduced alphabets, created using a Genetic Algorithm, allows an improvement over other state-of-the-art feature extraction methods.

**Conclusion:**

The validity of the novel strategy for creating reduced alphabets is demonstrated by the performance improvement obtained by the proposed approach with respect to other reduced alphabets-based methods in the tested problems.

## Background

In the literature several feature extraction approaches [1] have been proposed for the representation of peptides (e.g orthonormal encoding, *n*-grams, ...); some of them have been used for building ensembles of classifiers based on the perturbation of features (i.e. each classifier is trained using a different feature set). Nanni and Lumini in [2] proposed to build an ensemble of classifiers where each classifier is trained using a different physicochemical property of the amino acids, the selection of the best physicochemical properties to be combined is performed by Sequential Forward Floating Selection [3]; the same feature extraction is also used in [4] to train a machine learning approach for protein subcellular localization. A system for the recognition of T-cell epitopes is presented in [5] based on the combination of two Support Vector Machines (SVM). The first SVM is trained using the information on amino acid positions, while the second SVM is trained using information extracted from the sparse indicator vector and the BLOSUM50 matrix.

**Figure 1:**
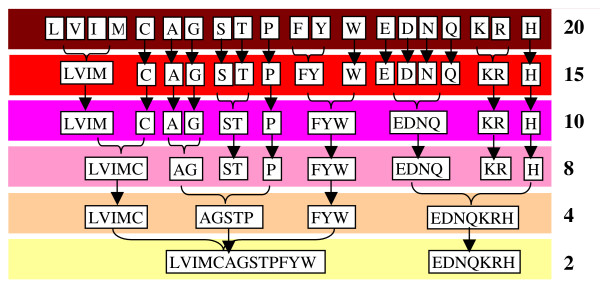
Schemes for reducing amino acid alphabet.

In particular, in [6] it is proposed an ensemble of SVM classifiers where each classifier is trained using a different *N*-peptide composition with reduced amino acid alphabets for larger values of *N*. The authors report that the ensemble of SVMs outperforms a stand-alone SVM trained using the well-known 2-peptide composition with the standard amino acid alphabet. In [6] the reduced alphabets are obtained in the following way: the 20-letter amino acid alphabet is reduced to smaller alphabets based on correlations indicated by the BLOSUM50 similarity matrix, i.e. amino acid pairs with high similarity scores are grouped together. The most correlated amino acids naturally form groups which have similar physiochemical properties (e.g. Hydrophobic residues, especially (LVIM) and (FYW), are conserved in many reduced alphabets, as they are the polar (ST), (EDNQ) and (KR) groups [7]). In Figure 1 the schemes, from [7], for reducing amino acid alphabet based on the BLOSUM50 matrix derived by grouping and averaging the similarity matrix elements are reported.

The complete group of reduced alphabets studied in [6] in addition to those delineated in Figure 1 are the following:

3 letters, [(LASGVTIPMC), (EKRDNQH), (FYW)];

5 letters, [(LVIMC), (ASGTP), (FYW), (EDNQ), (KRH)];

6 letters, [(LVIM), (ASGT), (PHC), (FYW), (EDNQ), (KR)];

12 letters, [(LVIM), (C), (A), (G), (ST), (P), (FY), (W), (EQ), (DN), (KR), (H)];

and 18 letters, [(LM), (VI), (C), (A), (G), (S), (T), (P), (F), (Y), (W), (E), (D), (N), (Q), (K), (R), (H)].

In this work an alternative way for building reduced alphabets is studied based on the use of Genetic Algorithm (GA) for grouping the amino-acids. The objective function of the Genetic Algorithm is the maximization of the area under the Receiver Operating Characteristic curve [5] for a given classification problem. In this way, several alphabets are created for a given value of their size. A different SVM [8] is trained on each feature set (each extracted from a different alphabet), finally this pool of classifiers is combined by the mean rule.

The approach proposed in this paper has been tested in three case studies: HIV-protease (two different datasets); recognition of T-cell epitopes; prediction of peptides that bind human leukocyte antigens.

AIDS is a grave, often mortal, disease of the immune system transmitted through HIV, therefore it is important to understand how HIV works. Some of the more successful drugs are HIV-1 protease inhibitors; in order to discover efficient HIV-1 protease inhibitors several automatic approaches have been developed aimed at obtaining a good understanding of the protease specificity (i.e., which peptides are cleaved by the HIV-1 protease and which are not). The standard paradigm for protease-peptide interactions is the "lock" and "key" model, where a sequence of amino acids fit as a "key" to the active site in the protease. The active site pockets of the protease are denoted by **S **which correspond to residues P in the peptide **P **= *P*_4_*P*_3_*P*_2_*P*_1_*P*_1_*'P*_2_*'P*_3_*'P*_4_*'*, where *P*_*i *_is an amino-acid belonging to Σ (Σ = {A,C,D....V,W,Y}). If the amino acids in **P **(the "key") fit the positions in **S **(the "lock"), then the protease cleaves the octamer between positions *P*_1 _and *P*_1_'.

Several works that try to solve the HIV-1 protease specificity problem by applying techniques from machine learning have been published [9-13]. Some methods based on a standard feed-forward multilayer perceptron are presented in [14,15]. In [9] it is shown that HIV-1 protease cleavage is a linear problem and that the best classifier for this problem is the Linear SVM. The interested reader can see [16] for a good review.

Antigenic peptides degraded from foreign or host proteins can bind to major histocompatibility complex (MHC) molecules. The major role MHC plays is to present the binding antigenic peptides to T-cell receptors (TCRs). Only when the TCRs recognize the antigen, the T-cell clone will be activated, and the cellular immune will happen. However, not all the MHC-peptide complexes can be recognized by TCRs. Those portions of short binding peptides, which can be recognized, are called T-cell epitopes [17]. Deciphering the patterns of peptides that elicit a MHC restricted T-cell response [5] is critical for vaccine development. Broadly, the methods developed to study the interaction between peptide and MHC are based on: structural information [18]; mathematical approaches including binding motifs [19]; quantitative matrices [20]; Artificial Neural Networks [21,22]; Support Vector Machines [23,5].

The prediction of peptides that bind multiple Human Leukocyte Antigen (HLA) molecules is crucial in the designing of vaccines that are useful to a broader population [24]. Several works have been developed for identification of HLA binding peptides, they include Support Vector Machines [25], Artificial Neural Networks [24], Hidden Markov Models [26]. These methods use the interaction (contact amino-acids) between peptides and HLA molecule to extract the features used to train the classifiers.

All the tests reported in this work have been conducted on 5 datasets: 2 HIV datasets (**HIV1 **and **HIV2), **a Peptide dataset for the recognition of T-cell epitopes **(PEP) **and two Vaccine Datasets (**VAC1 **and **VAC2); **please, see the Dataset sub-section of the Methods for a detailed description.

The GA optimization to find the best reduced alphabets is performed on two classification problems: HIV-protease (first dataset) and recognition of T-cell epitopes. Finally, these reduced alphabets are used in a second HIV dataset and in a third problem (the prediction of peptides that bind human leukocyte antigens); the experimental results demonstrate that, even if the reduced alphabets are not obtained on the same dataset, the performance in the HIV-protease and in the prediction of peptides that bind human leukocyte antigens improves with respect to that obtained by the state-of-the-art reduced alphabets-based feature extraction method.

Experimental results show that the novel multi-classifier approach outperforms the standard 2-peptide composition and the method proposed in [6] for all the three considered problems, demonstrating that the proposed method for producing reduced alphabets for peptide classification can be successfully applied to several bioinformatics problems.

## Results and Discussion

Among the independent dataset tests, sub-sampling test (e.g., 5 or 10-fold sub-sampling), and jackknife test, which are often used for examining the accuracy of a statistical prediction method, the jackknife test is deemed the most rigorous and objective as analyzed by a comprehensive review [27] and has been increasingly adopted by leading investigators to test the power of various prediction methods [28]. Anyway, in this work, due to computational issue, the testing results have been obtained using a 10-fold cross validation.

The fitness function of the Genetic Algorithm is the maximization of the Area Under the ROC-curve (AUC) using a leave one out on the training set for each dataset. The ROC-curve is a two-dimensional measure of classification performance that plots the probability of classifying correctly the positive examples against the rate of incorrectly classifying negative examples. AUC is also used for comparing classification performance; according to [29], AUC is preferred to accuracy (error rate), since it is statistically consistent and more discriminating than the accuracy measure. In fact, researchers are often interested in ranking of data samples rather than mere positive/negative classification results. Moreover, if class distribution is skewed or unbalanced, a classifier can still receive a high accuracy by simply classifying all data samples in the dominant class [30].

In the HIV datasets and in the Peptide dataset Linear SVM is used as stand-alone classifier, in the Vaccine datasets Radial Basis Function SVM is used. Notice that in both cases the parameters for SVM have not been optimized and they have been set to their default values (*C *= 1 and *Gamma *= 1). No parameter optimization has been performed in each dataset, since the aim of this work was to propose a generic method that could work well in several problems.

Tables 1 and 2 report the results of the proposed approach compared with a Baseline approach obtained considering the reduced alphabets yet proposed in the literature [6]. Several alphabets have been tested with different size *S *and *N*-peptide composition (see Section Methods): for the Baseline approach they refer to the reduced alphabets studied in [6] (see section 2), for the novel approach to the optimized alphabets. Notice that when the size *S *of the alphabet is 20 no reduction is carried out and all the approaches have the same performance (denoted by ") of Baseline. In the following, the novel approach will be denoted by GA(*K*)_*Set *_where *K *is the number of computation runs of the GA optimization (see Section Methods) and *Set *is the training set considered for the GA optimization. Possible values of *Set *are *H *= **HIV1**, *P *= **PEP**, *HP *= **HIV1+PEP **which means that alphabets are built considering both the datasets (the objective function of GA is the maximization of sum of two AUCs obtained in **HIV1 **and in **PEP**). The last two columns of Tables 1 and 2 denote the performance of the ensembles obtained by the fusion among the whole set ((*N *= 1, *S *= 20); (*N *= 2,*S *= 20); (*N *= 2,*S *= 8); (*N *= 1,*S *= 8); (*N *= 2,*S *= 4); (*N *= 1,*S *= 4)) of 6 alphabets (FUS1) and among the last 5 alphabets ((*N *= 2,*S *= 20); (*N *= 2,*S *= 8); (*N *= 1,*S *= 8); (*N *= 2,*S *= 4); (*N *= 1,*S *= 4)) (FUS2) by the sum rule.

**Table 1:** AUC obtained in the HIV1 dataset.

**HIV1 (AUC)**	**N = 1, S = 20**	**N = 2, S = 20**	**N = 2, S = 8**	**N = 1, S = 8**	**N = 2, S = 4**	**N = 1, S = 4**	**FUS1**	**FUS2**
**Baseline**	0.942	0.956	0.889	0.829	0.858	0.687	0.949	0.933
**GA(1)**_***H***_	"	"	0.894	0.871	0.878	0.820	0.938	0.940
**GA(5)**_***H***_	"	"	0.973	0.932	0.966	0.943	0.973	**0.977**
**GA(15)**_***H***_	"	"	0.980	0.909	0.957	0.889	0.953	0.965
**GA(5)**_***P***_	"	"	0.943	0.868	0.887	0.701	0.959	0.962
**GA(5)**_***HP***_	"	"	0.948	0.905	0.929	0.902	0.941	0.959
GA*(5)_***H***_	"	"	0.982	0.927	0.954	0.929	0.963	0.969

**Table 2:** AUC obtained in the PEP dataset.

**PEP (AUC)**	**N = 1, S = 20**	**N = 2, S = 20**	**N = 2, S = 8**	**N = 1, S = 8**	**N = 2, S = 4**	**N = 1, S = 4**	**FUS1**	**FUS2**
**Baseline**	0.855	0.887	0.795	0.830	0.906	0.890	0.908	0.890
**GA(5)**_***H***_	"	"	0.914	0.842	0.897	0.782	0.916	0.910
**GA(1)**_***P***_	"	"	0.859	0.855	0.914	0.823	0.930	0.919
**GA(5)**_***P***_	"	"	0.938	0.836	0.940	0.846	0.924	**0.949**
**GA(15)**_***P***_	"	"	0.925	0.891	0.937	0.841	0.933	0.924
**GA(5)**_***HP***_	"	"	0.928	0.785	0.844	0.812	0.934	0.944
GA*(5)_***P***_	"	"	0.945	0.865	0.951	0.871	0.911	0.917

In the last rows of Tables 1 and 2 other tests varying the parameters of the Genetic Algorithm (see Section Methods) are reported: GA*(*K*) denotes a variation of GA(*K*) where the number of chromosomes used by GA is *D *= 25 (instead of 10) and the number of generation steps is *E *= 10 (instead of 5).

From the analysis of the experimental results reported in Tables 1 and 2 for the datasets **HIV1 **and **PEP**, the following observations may be made:

- the method proposed in [6] outperforms the well known 2-peptide composition (*N *= 2, *S *= 20) in the **PEP **dataset but not in the **HIV1 **dataset;

- the new method outperforms both 2-peptide composition and [6] when *K *≥ 5.

- the performance of GA(*K*)_*HP *_is lower than GA(*K*)_*H*_, anyway it outperforms the standard 2-peptide composition and the method proposed in [6] in both the datasets.

- GA(5)_*H *_and GA(5)_*P *_work better than GA*(5)_*H *_and GA*(5)_*P *_in the **HIV1 **and **PEP **datasets, respectively; this behavior is probably due to the fact that GA*(5) is more overfitted on the validation set used to create the alphabets.

The groups of reduced alphabets generated by different runs of the Genetic Algorithm are not always the same, due to the stochasticity of the generation approach; anyway this cannot be considered a drawback since it permits to create an ensemble based on the perturbation of features. In the following a sample of reduced alphabets obtained by GA(1)_*HP *_is reported:

*N *= 2, *S *= 8, [(C), (EPQVW), (Y), (AFHILM), (DN), (), (RT), (KS)];

*N *= 1, *S *= 8, [(), (C), (GLNPQR), (ADHK), (EFIY), (T), (MV), (SW)];

*N *= 2, *S *= 4, [(DQY), (EFILPTV), (AGKMNRW), (CHS)];

*N *= 1, *S *= 4, [(GIN), (CKRSV), (AEFMPWY), (DHLQT)].

The variation among the alphabets obtained in different runs of the Genetic algorithm have been studied using the average Jaccard coefficient. The Jaccard coefficient [31] is a measure of the degree of similarity between two clusterings (i.e. two alphabets A and B) that is maximized if all the couples of patterns which belong to the same group in A, belong to the same group also in B:

*JAC *= *SS*/(*SS *+ *SD*)

where *SS *is the number of couples of amino acids that in both alphabets are grouped together and *SD *is the number of couples of amino acids that belong to the same group in one alphabet but not in the other. Table 3 reports the average Jaccard coefficient evaluated on 10 alphabets obtained by GA(1)_*HP*_, these results show that the alphabets are quite stable.

**Table 3:** Average Jaccard coefficient evaluated on 10 alphabets with different *N *and *S*.

**Alphabets**		**AVG(*JAC*)**
***N***	***S *(size)**	

1	8	0.79
2	8	0.78
1	4	0.65
2	4	0.66

**Figure 2:**
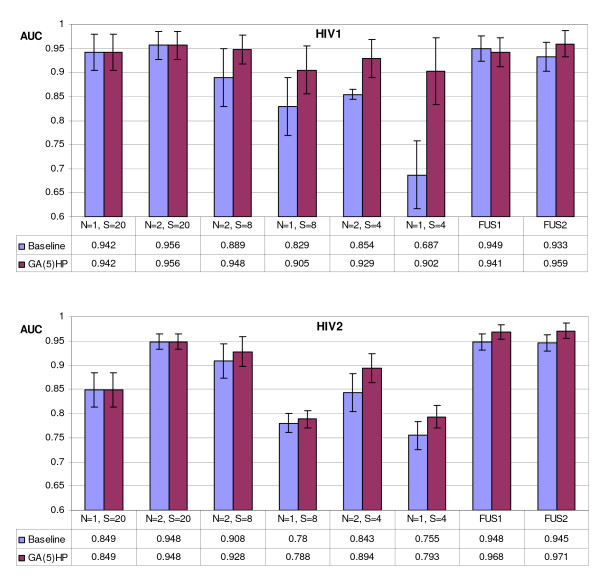
Comparison, in the HIV datasets, between the new GA(5)_*HP *_approach and the Baseline approach on all the 5 tested datasets. The Error bars represent the standard deviation of the mean.

**Figure 3:**
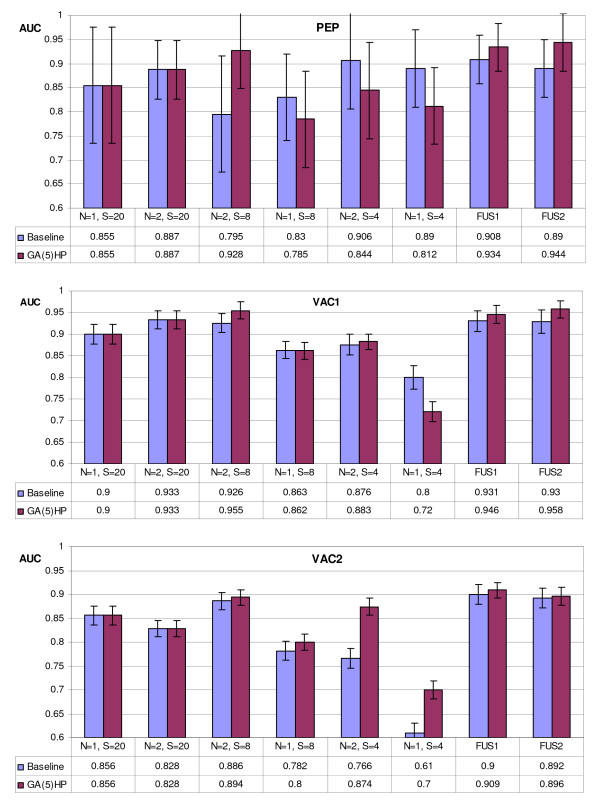
Comparison, in the other datasets, between the new GA(5)_*HP *_approach and the Baseline approach on all the 5 tested datasets. The Error bars represent the standard deviation of the mean.

In Figures 2, 3 the graphs showing the AUC gained by the GA(5)_*HP *_approach and the Baseline approach on all the 5 tested datasets are reported. GA(*5*)_*HP *_outperforms the approaches obtained with the other *N*-peptide composition based feature extractions also in the datasets not used for the optimization of the reduced alphabets; these tests are a further demonstration of the importance of building an ensemble of classifiers perturbing the feature set. The error bars in Figure 2, 3, representing the standard deviation of the mean, show that GA(*5*)_*HP *_is slightly more stable than Baseline.

In Table 4 the error rates related to the approaches compared above are reported. Even if AUC is a more robust measure for comparing classifiers, it could be interesting to compare methods also in term of accuracy/error rate. The results of the new approach in terms of error rate are not as good as in terms of AUC, anyway it should be noted that the new ensemble has not been optimized to minimize the error rate.

**Table 4:** Error Rate in the 5 datasets.

**Error Rate**		**N = 1, S = 20**	**N = 2, S = 20**	**N = 2, S = 8**	**N = 1, S = 8**	**N = 2, S = 4**	**N = 1, S = 4**	**FUS1**	**FUS2**
**HIV1**	**Baseline**	0.133	0.105	0.187	0.295	0.211	0.339	0.153	0.156
	**GA(5)**_***HP***_	0.133	0.105	0.117	0.165	0.117	0.21	0.105	**0.100**
**HIV2**	**Baseline**	0.145	**0.054**	0.107	0.219	0.179	0.219	0.153	0.157
	**GA(5)**_***HP***_	0.145	**0.054**	0.137	0.24	0.208	0.24	0.147	0.149
**PEP**	**Baseline**	0.215	0.17	0.225	0.215	0.215	0.215	0.175	0.175
	**GA(5)**_***HP***_	0.215	0.17	**0.120**	0.15	0.15	0.15	0.15	0.15
**VAC1**	**Baseline**	0.120	0.12	0.114	0.15	0.14	0.16	0.131	0.129
	**GA(5)**_***HP***_	0.120	0.12	0.108	0.188	0.187	0.188	**0.107**	0.110
**VAC2**	**Baseline**	0.210	0.17	0.173	0.3	0.285	0.311	0.285	0.275
	**GA(5)**_***HP***_	0.210	0.17	0.17	0.31	0.2515	0.310	**0.15**	**0.15**

**Figure 4:**
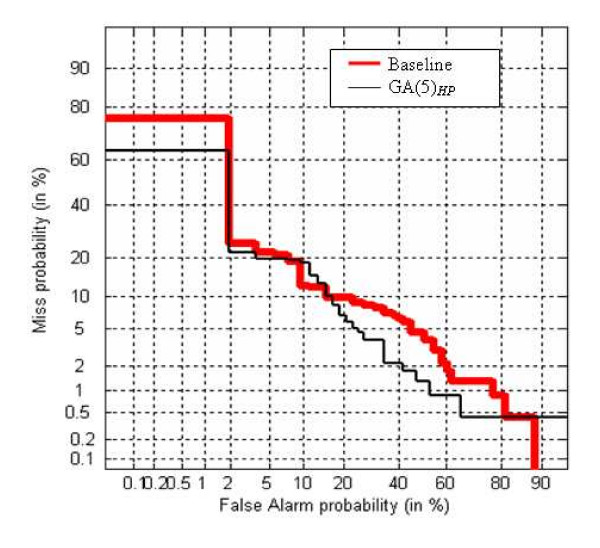
DET-curve for FUS2 on the **VAC1 **dataset.

**Figure 5:**
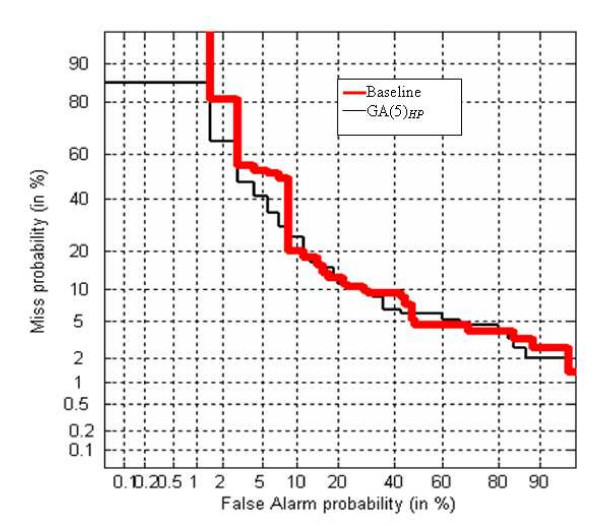
DET-curve for FUS1 on the **VAC2 **dataset.

Finally, in order to confirm the benefit of the novel alphabet generation with respect to the Baseline approach, the DET curve has been plotted. The DET curve [32] is a two-dimensional measure of classification performance that plots the probability of false acceptation against the rate of false rejection. In Figure 4 the DET curve obtained by FUS2 is plotted varying the alphabets (Baseline and GA(5)_*HP*_) for **VAC1 **dataset. In Figure 5 the DET curve obtained by FUS1 for **VAC2 **dataset is plotted.

## Conclusion

In this paper, it is proposed a new algorithm which uses a series of Support Vector Machines in conjunction with a set of reduce alphabets of the amino-acids to obtain a novel multi-classifier based on the perturbation of features, where each classifier is trained using a different reduced alphabet. The reduced alphabets are generated using a novel approach based on Genetic Algorithm whose objective function is the maximization of the AUC obtained in several classification problems. The alphabets creation problem can be viewed as a clusterization problem: the Genetic Algorithm is suited for this purpose since it does not need a vectorial representation of the amino-acid and permits an ad-hoc search based on an appropriate fitness function; therefore the resulting alphabets are optimized for the considered classification problem. Of course, several other meta-heuristic approaches (e.g. Particle Swarm Optimization, Ants Systems, ...) could be tested for the same aim.

The validity of the novel strategy for the generation of reduced alphabets is demonstrated by the performance improvement obtained by the proposed approach with respect to another reduced alphabets-based method in the tested problems. The importance of the encoding based on reduced alphabets goes over the performance of the proposed approach, and can be related to the possibility of creating an ensemble based on methods that use different feature extractions. In the literature [1], it has been clearly shown that the fusion of classifiers based on different feature encodings permits to obtain a large error reduction with respect to the performance of a stand-alone method.

## Methods

### Datasets

The tests have been conducted on the following 5 datasets:

#### HIV datasets

The two datasets contains octamer protein sequences, each of which needs to be classified as an HIV protease cleavable site or uncleavable site. **HIV1 **[9,2] contains 362 octamer protein sequences (114 cleavable and 248 uncleavable), while **HIV2 **[33] (which includes **HIV1**) contains 1625 octamer protein sequences (374 cleavable and 1251 uncleavable).

#### Peptide dataset (PEP)

This dataset contains 203 synthetic peptides and it is the same used in [23,2]. Peptides were synthesized by the simultaneous-multiplepeptide-synthesis methods and characterized using HPLC and mass spectrometry.

#### Vaccine Datasets

The datasets employed in [24-26] are used, performing blind testing on five HLA-A2 and seven HLA-A3 molecules. The predictive accuracy of peptide binding is tested separately to HLA-A2 (**VAC1**) and HLA-A3 (**VAC2**) variants. **VAC1 **contains 3041 samples (664 belong to the class Binders), while **VAC2 **contains 2216 samples (680 belong to the class Binders).

### Alphabets creation

**Figure 6:**
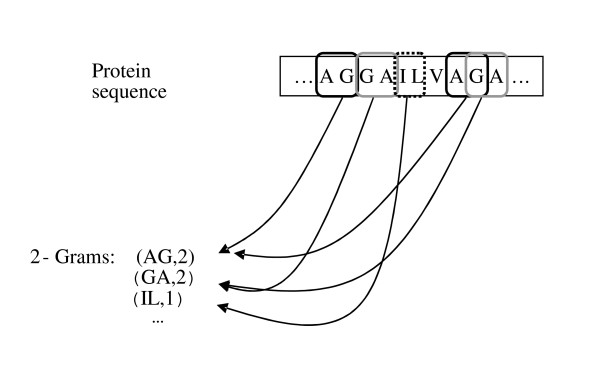
An example of 2-Grams (from [3]).

In the *N*-peptide composition for each value of *N*, the corresponding feature vector contains the fraction of each possible *N*-length substring in the sequence. Therefore the feature vector refers to amino acid composition for *N *= 1 and dipeptide composition for *N *= 2. The number of dimensions in the feature vector corresponding to n-peptide composition is 20^*N*^. An example of 2-grams is shown in Figure 6 (from [3]).

The main problem is that for large values of *N *the dimension of the feature set is unfeasible, for this reason in [6] reduced amino acid alphabets are used for training the classifiers with a *N*-peptide composition (*N*>2). In [6] the authors propose their method for extracting features from proteins, in this paper the features are extracted from peptides (each pattern is less than 10 amino-acids), hence a *N*-peptide composition with *N*>2 is not useful. The resulting reduced alphabets are used for building an ensemble of classifiers based on the perturbation of the feature set [3].

**Figure 7:**
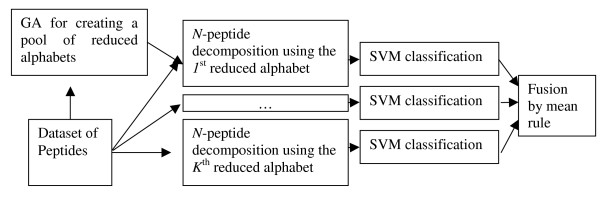
Block-diagram of the proposed system.

In this work, an alternative way for the construction of reduced alphabets is studied, based on a Genetic Algorithm for grouping amino-acids. The objective function of the Genetic Algorithm is the maximization of the AUC for a given classification problem. *K *different alphabets are created for each value of the size *S *of the reduced alphabets and for a given value of *N*. The *i*^*th *^reduced alphabet is built considering the previous reduced alphabets of the same size *S *and of the same value of *N*. Simply, for the calculation of the objective function of the *i*^*th *^iteration of GA the scores obtained by the *i*^*th *^reduced alphabet are combined by the mean rule with the scores obtained by the previous *i-*1 reduced alphabets. The mean rule selects as final score (*score*(**s**,c)) the mean score of a pool of *K *classifiers.

$$score(s,c)=\frac{1}{K}\sum_{j=1\ldots K}sim_{j}(s,c)$$

where *sim*_*j*_(**s**,*c*) is the similarity of the pattern **s **to the class *c*, obtained by the *j*^*th *^classifier. The block-diagram of the proposed system is shown in Figure 7.

Table 5 reports the amino acid alphabet sizes and the resulting feature vector dimensions used for the peptide compositions tested in this paper.

**Table 5:** The Amino Acid Alphabet Sizes and Resulting Feature Vector Dimensions Used for *N*-Peptide Compositions.

***N***	**Size reduced alphabet (*S*)**	**Feature Vector Dimension**
1	20	20
2	20	400
1	8	8
2	8	64
1	4	4
2	4	16

### Genetic algorithm

Genetic Algorithms (Implemented as in GAOT MATLAB TOOLBOX) are a class of optimization methods inspired by the process of the natural evolution [34]. These algorithms operate iteratively on a population of chromosomes, each of which represents a candidate solution to the problem.

In the encoding scheme, the chromosome **C **is a string whose length is 20 (the number of amino-acids). Each value in the chromosome specifies at which group a given amino-acid belongs. Notice that it is not checked if a group is empty, therefore in a reduced alphabet of dimension *S *it is possible that some groups are empty.

The initial population is a randomly generated set of chromosomes, then a fixed number *E *(in this paper *E *= 5) of generation steps is performed by the application of the following basic operators: selection, crossover and mutation.

#### Selection

The selection strategy is cross generational. Assuming a population of size *D *(in this paper *D *= 10), the offspring doubles the size of the population and the best *D *individuals from the combined parent-offspring population are retained.

#### Crossover

Uniform crossover is used, the crossover probability is fixed to 0.96 in the experiments.

#### Mutation

The mutation probability is 0.02.

## Authors' contributions

LN designed and implemented the algorithms. AL coordinated the study. LN and AL write, read and approved the final manuscript.
